# Kinetics of H_2_O_2_-driven catalysis by a lytic polysaccharide monooxygenase from the fungus *Trichoderma reesei*

**DOI:** 10.1016/j.jbc.2021.101256

**Published:** 2021-09-28

**Authors:** Silja Kuusk, Priit Väljamäe

**Affiliations:** Institute of Molecular and Cell Biology, University of Tartu, Tartu, Estonia

**Keywords:** lytic polysaccharide monooxygenase, hydrogen peroxide, peroxygenase, peroxidase, enzyme inactivation, ABTS, 2,2′-azino-bis(3-ethylbenzothiazoline-6-sulfonic acid), AscA, ascorbic acid/ascorbate, BMCC, bacterial microcrystalline cellulose, Glc_eq_, glucose equivalents, GO, glucose oxidase, HRP, horseradish peroxidase, LPMO, lytic polysaccharide monooxygenase

## Abstract

Owing to their ability to break glycosidic bonds in recalcitrant crystalline polysaccharides such as cellulose, the catalysis effected by lytic polysaccharide monooxygenases (LPMOs) is of major interest. Kinetics of these reductant-dependent, monocopper enzymes is complicated by the insoluble nature of the cellulose substrate and parallel, enzyme-dependent, and enzyme-independent side reactions between the reductant and oxygen-containing cosubstrates. Here, we provide kinetic characterization of cellulose peroxygenase (oxidative cleavage of glycosidic bonds in cellulose) and reductant peroxidase (oxidation of the reductant) activities of the LPMO *Tr*AA9A of the cellulose-degrading model fungus *Trichoderma reesei*. The catalytic efficiency $\left(k_{\text{cat}}/K_{\text{m}\left({\text{H}}_{2}{\text{O}}_{2}\right)}\right)$ of the cellulose peroxygenase reaction (*k*_cat_ = 8.5 s^−1^, and $K_{\text{m}\left({\text{H}}_{2}{\text{O}}_{2}\right)}=30\;\text{μM}$) was an order of magnitude higher than that of the reductant (ascorbic acid) peroxidase reaction. The turnover of H_2_O_2_ in the ascorbic acid peroxidase reaction followed the ping-pong mechanism and led to irreversible inactivation of the enzyme with a probability of 0.0072. Using theoretical analysis, we suggest a relationship between the half-life of LPMO, the values of kinetic parameters, and the concentrations of the reactants.

Lytic polysaccharide monooxygenases (LPMOs) are monocopper enzymes that catalyze oxidative cleavage of glycosidic bonds in various polysaccharides (1, 2, 3, 4, 5). LPMOs are widespread in nature, and they are classified within several auxiliary activity families in the database of carbohydrate-active enzymes (6). Although structurally and biochemically well characterized (7, 8), the kinetic data of LPMOs are scarce. Besides polysaccharide substrate and oxygen-containing cosubstrate (O_2_/H_2_O_2_), LPMOs need a reductant for their activity (9, 10, 11, 12). In H_2_O_2_-driven oxidative cleavage of glycosidic bond (polysaccharide peroxygenase reaction), the reductant is needed only for the initial priming reduction of LPMO-Cu(II) resting state to a catalytically active LPMO-Cu(I) (12). LPMO-Cu(I) activates H_2_O_2_ leading to the hydroxylation and cleavage of glycosidic bond (Fig. 1) (12). When the LPMO active site is free from polysaccharide, the LPMO-Cu(I) can be reoxidized by H_2_O_2_ to LPMO-Cu(II). The latter results in the stoichiometric oxidation of the reductant and is referred to as the reductant peroxidase reaction (Fig. 1). Kinetic studies of LPMOs are complicated by the insoluble nature of the substrate, various side reactions such as reductant oxidase/peroxidase activity of LPMOs, and inactivation of LPMOs in reductant peroxidase reaction (13). Furthermore, an enzyme-independent oxidation of the reductant by O_2_ often leads to the formation of H_2_O_2_ (14, 15). These multiple parallel side reactions not only complicate the analysis of the kinetic data but may also lead to wrong conclusions regarding the nature of the cosubstrate as exemplified by the LPMO of the bacterium *Serratia marcescens* (*Sm*AA10A), which was first identified as a monooxygenase (1) but later turned out to be a peroxygenase (16). An analogous example is provided by a fosfomycin-producing non–heme iron epoxidase that was initially identified as an oxidase (17) but turned out to be a peroxidase (18). Despite the major interest, to date, a detailed kinetic characterization is available only for the O_2_-driven oxidation of soluble cellohexaose by an LPMO from the fungus *Myceliophthora thermophila* (*Mt*PMO9E) (19), the H_2_O_2_-driven oxidation of insoluble chitin by *Sm*AA10A (20) and soluble chitooligosaccharides by *Af*AA11B from the fungus *Aspergillus fumigatus* (21). In a recent study, several LPMOs were also characterized in terms of catalytic efficiency $\left(k_{\text{cat}}/K_{\text{m}\left({\text{H}}_{2}{\text{O}}_{2}\right)}\right)$ of the cellulolytic peroxygenase reaction (22). Since the discovery of the peroxygenase activity in 2017 (16), a number of further studies have reported much higher rates with H_2_O_2_ in the oxidation of both, the substrate (15, 20, 21, 22, 23, 24, 25, 26, 27, 28, 29, 30) and the reductant (28, 31, 32, 33, 34, 35). The results of computational studies also suggest that H_2_O_2_ is a relevant cosubstrate of LPMOs (33, 36). Because of the great potential in various applications, the activity of LPMOs on cellulose is of particular interest (37). Enzymatic degradation of lignocellulose takes place in the complex redox-active environment (38, 39) that can provide LPMOs with electrons and the H_2_O_2_ cosubstrate (26). However, the lack of the in-depth knowledge on the kinetics of LPMO catalysis hampers rational fine-tuning of the reaction conditions toward optimal activity and stability of the LPMOs.

**Figure 1:**
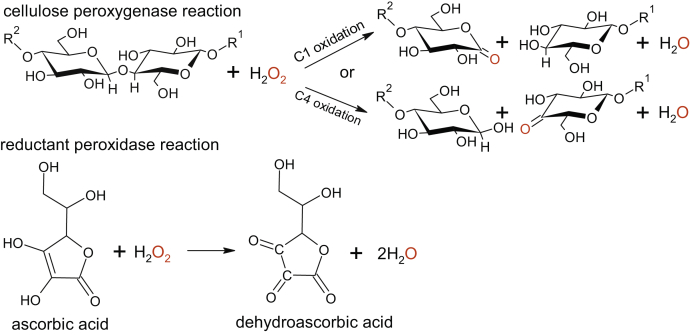
**H**_**2**_**O**_**2**_**-driven catalysis by LPMO involves cellulose peroxygenase and reductant peroxidase reactions.** Cellulose peroxygenase reaction takes place with the Cu(I) form of LPMO. The enzyme-independent hydration of oxidized sugar products to aldonic acid and 4-gemdiol, for C1 and C4 oxidized sugars, respectively, is not shown. R^1^ and R^2^ stand for the reducing- and nonreducing-end sides of the cellulose chain, respectively. Reductant (shown using ascorbic acid as an example) peroxidase reaction takes place with the Cu(II) form of LPMO. LPMO, lytic polysaccharide monooxygenase.

Here, we used a uniformly ^14^C-labeled cellulose substrate that enabled kinetic characterization of the H_2_O_2_-driven cellulose peroxygenase reaction by the LPMO (*Tr*AA9A) of the model fungus *Trichoderma reesei* (also known as *Hypocrea jecorina*). The catalytic efficiency of the cellulose peroxygenase reaction (2.9 × 10^5^ M^−1^ s^−1^) was an order of magnitude higher than that of the reductant (ascorbate, AscA) peroxidase reaction. The AscA peroxidase reaction also led to the irreversible inactivation of *Tr*AA9A with the probability of 0.0072.

## Results

### Kinetics of cellulolytic peroxygenase reaction

The use of a ^14^C-labeled polysaccharide substrate enables sensitive detection of soluble LPMO products (20). Here, we provide a kinetic characterization of the cellulolytic peroxygenase reaction of an LPMO using uniformly ^14^C-labeled bacterial microcrystalline cellulose (BMCC) as a substrate and *Tr*AA9A as a model LPMO. *Tr*AA9A has been shown to release both C1 and C4 oxidized sugar products from cellulose (40, 41, 42). Before we describe the results, we note that, provided with the value of the stoichiometry showing the amount of the released soluble products per molecule of H_2_O_2_ consumed, the regioselectivity of oxidation and the distribution of products between the soluble and the insoluble fractions are not important in deriving the values of kinetic parameters (20).

In the presence of the reductant, ascorbic acid (AscA), and H_2_O_2_, *Tr*AA9A released ^14^C-labeled soluble products from BMCC (Fig. S1). No release of radioactivity was observed in the absence of added H_2_O_2_ (Fig. S1), suggesting that the possible *Tr*AA9A monooxygenase reaction and the production of H_2_O_2_ in *Tr*AA9A-dependent (AscA oxidase) and *Tr*AA9A-independent reactions are insignificant under our study conditions. The rate of the release of soluble products (expressed in glucose equivalents, Glc_eq_) increased with the increasing concentration of AscA (Fig. S2). The apparent half-saturating concentration of AscA was around 0.1 mM, and based on these results, we choose 1 mM AscA in the further experiments of BMCC degradation.

**Figure 2:**
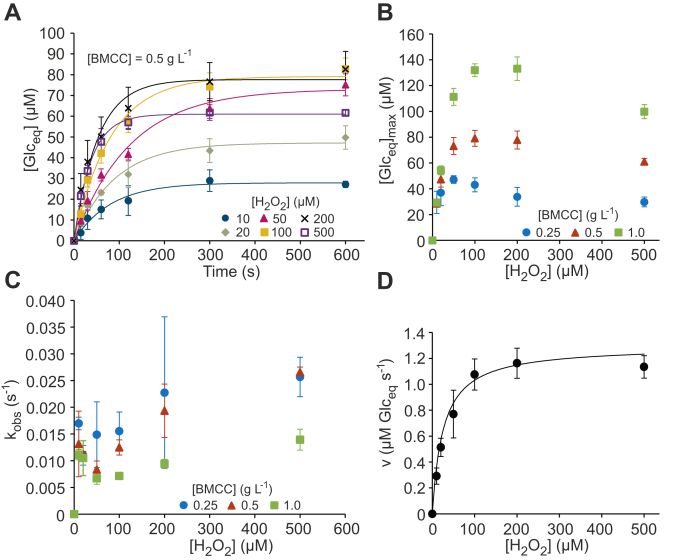
**Kinetics of the cellulose (BMCC) peroxygenase reaction.***A*, progress *curves* of the release of soluble products (expressed in glucose equivalents, Glc_eq_) from BMCC (0.5 g l^−1^) by *Tr*AA9A (50 nM) at different concentrations of H_2_O_2_ (indicated in the plot). The reactions were made in sodium acetate (50 mM, pH 5.0) at 25 °C in the presence of 1 mM AscA. The *solid lines* show nonlinear regression of the data according to Equation 1. For the progress *curves* made with 0.25 and 1.0 g l^−1^ BMCC, refer Fig. S3. *B*, dependency of [Glc_eq_]_max_ on the concentration of H_2_O_2_. *C*, dependency of *k*_obs_ on the concentration of H_2_O_2_. The concentration of cellulose is indicated in the plot. The [Glc_eq_]_max_ and *k*_obs_ values were derived from nonlinear regression of the progress *curves* according to Equation 1. *D*, dependency of initial rates of the release of soluble products on the concentration of H_2_O_2_. The *solid line* shows nonlinear regression of the data according to the Michaelis–Menten equation. Shown are the average values and SD from the experiments made with 0.5, 1.0, and 1.5 g l^−1^ BMCC (refer to Fig. S4*A* to see them separately). In panels *A*–*C*, error bars show ±SD (*n* = 3, independent experiments). AscA, ascorbic acid/ascorbate; BMCC, bacterial microcrystalline cellulose.

Progress curves of the release of soluble Glc_eq_ from BMCC by *Tr*AA9A at different [H_2_O_2_] are shown in Figure 2*A*. Single exponential function was used as a first approximation in the analysis of the progress curves (20).

(1) $$\left[{\text{Glc}}_{\text{eq}}\right]={\left[{\text{Glc}}_{\text{eq}}\right]}_{\text{max}}\left(1-e^{-k_{\text{obs}}t}\right)$$

In Equation 1, [Glc_eq_] is the concentration of soluble products (μM), [Glc_eq_]_max_ is the maximum concentration of Glc_eq_ that is released under given experiment conditions, and *k*_obs_ is the observed first-order rate constant (s^−1^). [Glc_eq_]_max_ first increased with increasing [H_2_O_2_] but started to decrease at H_2_O_2_ concentrations above 50 μM (Fig. 2*B*). On the other hand, the initial decrease of *k*_obs_ with [H_2_O_2_] was followed by its increase at [H_2_O_2_] above 50 μM (Fig. 2*C*). These kinetic signatures are similar to those observed at the H_2_O_2_-driven degradation of chitin by *Sm*AA10A and suggest that the irreversible inactivation of LPMO takes place in parallel with the polysaccharide peroxygenase reaction (20). At low [H_2_O_2_], the H_2_O_2_ is depleted in the cellulose peroxygenase reaction, and [Glc_eq_]_max_ represents the maximum number of soluble products that can be released by the given amount of H_2_O_2_. At high [H_2_O_2_], the enzyme is inactivated (with the half-life of ln2/*k*_obs_) before H_2_O_2_ is depleted in the cellulose peroxygenase reaction (20).

The dependency of the initial rates of Glc_eq_ formation on [H_2_O_2_] was consistent with the Michaelis–Menten equation (Fig. 2*D*). The Michaelis–Menten curves measured at BMCC concentrations of 0.5, 1.0, and 1.5 g l^−1^ overlapped within experiment scatter, indicating that the enzyme was saturated with cellulose (Fig. S4*A*). Measurements of the concentration of the cellulose-free *Tr*AA9A also suggested strong binding to BMCC (Fig. S5) in the presence of 1 mM AscA. The binding was significantly weaker in the absence of AscA (Fig. S5). High affinity to BMCC does not allow precise measurement of initial rates at the subsaturating BMCC concentrations (*i.e.*, very low), and thus, we cannot assess the dependency of apparent *V*_max_ and $K_{\text{m}\left({\text{H}}_{2}{\text{O}}_{2}\right)}$ values on [BMCC] (Fig. S4, *B*–*D*). Using an average value of initial rates measured at BMCC concentrations of 0.5, 1.0, and 1.5 g l^−1^ (Fig. 2*D*), we estimated the true *V*_max_ and $K_{\text{m}\left({\text{H}}_{2}{\text{O}}_{2}\right)}$ values of 1.28 ± 0.05 μM Glc_eq_ s^−1^ and 30 ± 5 μM, respectively. Considering the total concentration of *Tr*AA9A of 0.05 μM, *V*_max_ translates to the *k*_cat_ value of 25.6 ± 1.0 soluble Glc_eq_ s^−1^. To find the *k*_cat_ value for the cellulolytic peroxygenase reaction, one must know the value of the stoichiometry coefficient (*n*), which shows an average number of soluble products (in Glc_eq_) released per molecule of H_2_O_2_ consumed in the peroxygenase reaction. The value of *n* can be found from the [Glc_eq_]_max_ values measured under conditions that favor the cellulose peroxygenase reaction, that is, at low [H_2_O_2_] and high [BMCC]. The [Glc_eq_]_max_ values measured using 10 μM H_2_O_2_ were independent of [BMCC] (Fig. 2*B*), and based on these figures, we estimated the value of *n* = 2.90 ± 0.05 soluble Glc_eq_/H_2_O_2_. We also used an alternative approach for the determination of the value of *n*, where H_2_O_2_ was *in situ* produced by the glucose/glucose oxidase (GO) reaction with the rate of 1.2 ± 0.1 μM min^−1^ (Fig. S6*A*). The time curves of Glc_eq_ formation were independent of [*Tr*AA9A], indicating that the rate is limited by the GO reaction but deviated from linearity with the effect being larger at lower [BMCC] (Fig. S6*B*). However, at the shortest incubation time (10 min), the rate of Glc_eq_ formation was independent of [BMCC], and based on these data, we estimated the value of *n* = 3.1 ± 0.2 soluble Glc_eq_/H_2_O_2_ (Fig. S6*B*). Combining the *n* values measured using two different approaches results in an average *n* value of 3.0 ± 0.15 soluble Glc_eq_/H_2_O_2_ for the *Tr*AA9A/BMCC system. Provided with the value of *n*, we can now calculate the *k*_cat_ value of 8.5 ± 0.4 s^−1^ and $k_{\text{cat}}/K_{\text{m}\left({\text{H}}_{2}{\text{O}}_{2}\right)}$ value of 290,000 ± 50,000 M^−1^ s^−1^ for the cellulolytic peroxygenase reaction of *Tr*AA9A (Table 1).

**Table 1:** Kinetic parameters of the cellulose peroxygenase and the ascorbate peroxidase reactions of *Tr*AA9A

Kinetic parameter	Reaction^a^	
	Cellulose peroxygenase	Ascorbate peroxidase
*k*_cat_ (s^−1^)	8.5 ± 0.4	2.1^b^
$K_{\text{m}\left({\text{H}}_{2}{\text{O}}_{2}\right)}\left(\text{μM}\right)$	30 ± 5	78^b^
$k_{\text{cat}}/K_{\text{m}\left({\text{H}}_{2}{\text{O}}_{2}\right)}\left({\text{M}}^{-1}\;{\text{s}}^{-1}\right)$	290,000 ± 50,000	26,900 ± 3000
Probability of inactivation (*P*_i_)		0.0072 ± 0.0003
$k_{\text{cat}}/K_{\text{m}\left({\text{H}}_{2}{\text{O}}_{2}\right)}$ of inactivation (M^−1^ s^−1^)		195 ± 23

a All reactions were made at pH 5 and 25 °C.

b Because of insufficient saturation with AscA, these figures must be treated with caution.

### Kinetics of reductant peroxidase reaction

Decreasing [Glc_eq_]_max_ values of the peroxygenase reaction with decreasing [BMCC] already at H_2_O_2_ loads below the $K_{\text{m}\left({\text{H}}_{2}{\text{O}}_{2}\right)}$ of the peroxygenase reaction (see results with 20 μM H_2_O_2_ in Fig. 2*B*) indicates that H_2_O_2_ is also consumed in other reaction(s). The reductant peroxidase reaction of LPMOs is a well-known side reaction that involves the reoxidation of reduced LPMO-Cu(I) by H_2_O_2_ (11, 33, 34). Because the peroxidase reaction takes place with the population of LPMOs with the active site free from the substrate, its contribution is expected to increase with the decreasing concentration of the polysaccharide substrate. Importantly, the peroxidase reaction also leads to the irreversible inactivation of LPMO (16). Therefore, we also performed an in-depth kinetic characterization of the AscA peroxidase reaction of *Tr*AA9A.

**Figure 3:**
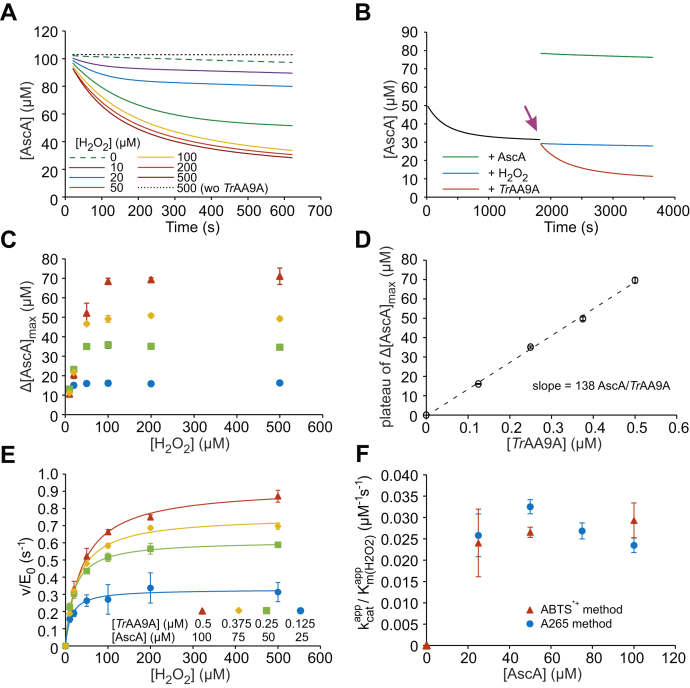
**Kinetics of the reductant (AscA) peroxidase reaction.***A*, progress *curves* of the oxidation of AscA (100 μM) by *Tr*AA9A (0.5 μM) at different concentrations of H_2_O_2_ (indicated in the plot). The reactions were made in sodium acetate (50 mM, pH 5.0) at 25 °C. For the progress *curves* made with other concentrations of AscA and *Tr*AA9A, refer Fig. S8. *B*, the oxidation of AscA (50 μM) by *Tr*AA9A (0.125 μM) in the presence of H_2_O_2_ (200 μM). After 1800 s (indicated with *arrow*), the reactions were supplemented with a new portion (in the concentration equivalent to the initial one) of *Tr*AA9A, AscA, or H_2_O_2_ (as indicated in the plot). *C*, the dependency of Δ[AscA]_max_ on the concentration of H_2_O_2_. The Δ[AscA]_max_ values were derived from nonlinear regression of the progress *curves* according to Equation 2. The concentration of AscA and *Tr*AA9*A* is defined in panel *E*. *D*, the dependency of the plateau value of Δ[AscA]_max_ (found from the data in panel *B* as an average Δ[AscA]_max_ at [H_2_O_2_] = 100, 200, and 500 μM) on the concentration of *Tr*AA9A. The *solid line* shows linear regression of the data. *E*, the dependency of the initial rates of AscA oxidation (divided by the concentration of *Tr*AA9A) on the concentration of H_2_O_2_. The *solid lines* show nonlinear regression of the data according to the Michaelis–Menten equation. The concentration of AscA and *Tr*AA9*A* is defined in the plot. *F*, the dependency of the apparent $k_{\text{cat}}/K_{\text{m}\left({\text{H}}_{2}{\text{O}}_{2}\right)}$ of the AscA peroxidase reaction on the concentration of AscA. One series in panel *F* show the results of the experiments obtained using the reduction of ABTS^•+^ for the detection of AscA (*n* = 3, independent experiments). Other results in this figure were obtained using the absorbance at 265 nm for the detection of AscA. Shown are the average values and ±SD (n = 2, independent experiments). For clarity, the SD are not shown for the traces in panels *A* and *B*. ABTS, 2,2′-azino-bis (3-ethylbenzothiazoline-6-sulfonic acid); AscA, ascorbic acid/ascorbate.

Figure 3*A* shows the time curves of AscA (100 μM) oxidation (followed by the absorbance at 265 nm, Fig. S7*A*) by 0.5 μM *Tr*AA9A at different initial H_2_O_2_ loads. Without *Tr*AA9A, AscA was stable even in the experiments with the highest (500 μM) H_2_O_2_ load (Figs. 3*A* and S8). A slow conversion of AscA was observed in the experiments without added H_2_O_2_ but in the presence of *Tr*AA9A (Figs. 3*A* and S8). In the experiments with *Tr*AA9A and H_2_O_2_, the [AscA] decreased exponentially to a nonzero plateau value (Fig. 3*B*). Supplementation of the reaction at this point with *Tr*AA9A resulted in a new burst of the AscA oxidation, whereas the supplementation of the reaction with AscA or H_2_O_2_ had no effect on the further rate of AscA oxidation (Fig. 3*B*). These results indicate that *Tr*AA9A has been inactivated before the complete consumption of AscA and/or H_2_O_2_. Equation 2 was used as the first approximation in the analysis of the time curves of AscA oxidation.

(2) $$\left[\text{AscA}\right]=\;\Delta {\left[\text{AscA}\right]}_{\text{max}}e^{-k^{\text{app}}t}+{\left[\text{AscA}\right]}_{\infty }$$

Δ[AscA]_max_ is the maximum concentration of AscA consumed in the reaction, *k*^app^ is the apparent first-order rate constant, and [AscA]_∞_ is the remaining concentration of AscA. At low [H_2_O_2_], Δ[AscA]_max_ increased with increasing [H_2_O_2_] but remained constant starting at 100 μM [H_2_O_2_] (Fig. 3*C*). This suggests that, at low initial H_2_O_2_ loads, Δ[AscA]_max_ is determined by the depletion of H_2_O_2_, whereas at high [H_2_O_2_], *Tr*AA9A is inactivated before AscA and H_2_O_2_ are consumed. An average value of Δ[AscA]_max_ measured in the experiments with low (10 μM) [H_2_O_2_] was 11.7 ± 1.1 (Fig. 3*C*), suggesting 1/1 stoichiometry in the AscA peroxidase reaction. The plateau values of Δ[AscA]_max_ (measured at [H_2_O_2_] of 100 μM and above) measured in the experiments with different [AscA] and [*Tr*AA9A] (to avoid full oxidation of AscA, the ratio of [AscA]/[*Tr*AA9A] was kept 200/1 in these experiments) scaled linearly with [*Tr*AA9A] with the slope equal to 138 ± 5 molecules of AscA oxidized per molecule of *Tr*AA9A (Fig. 3*D*). This figure translates to the probability of irreversible inactivation *P*_i_ = 0.0072 ± 0.0003 of *Tr*AA9A in the ascorbate peroxidase reaction (Table 1). We also analyzed the time curves of AscA peroxidase reaction after compensating them for the AscA oxidized in the absence of added H_2_O_2_ (Fig. S9). After compensation, Δ[AscA]_max_ measured in the experiments with 10 μM H_2_O_2_ was reduced to 9.1 ± 0.3, and the estimate of AscA oxidized per molecule of *Tr*AA9A was 130 ± 11 (*P*_i_ = 0.0077, Fig. S9).

**Figure 4:**
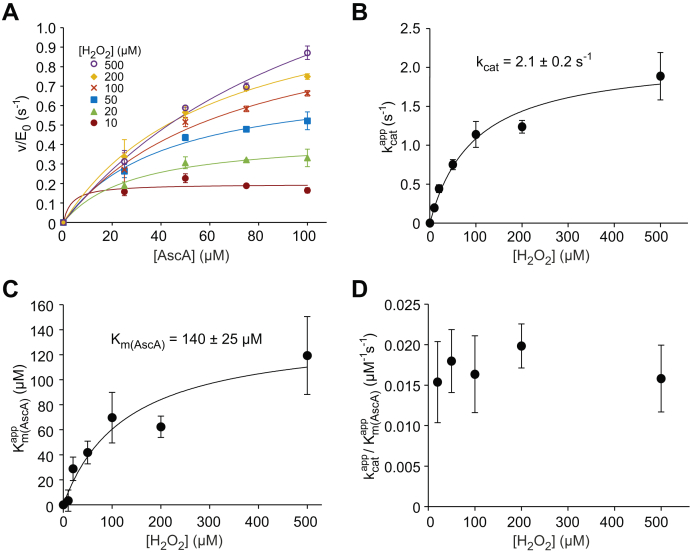
**Kinetic parameters of AscA peroxidase reaction.***A*, dependency of the initial rates of the oxidation of AscA (divided to the concentration of *Tr*AA9A) on the concentration of AscA. The concentration of H_2_O_2_ is indicated in the plot. The concentration of AscA was measured by measuring the absorbance at 265 nm. The *solid line* shows nonlinear regression of the data according to the Michaelis–Menten equation. Dependency of apparent (*B*) *k*_cat_, (*C*) *K*_m(AscA)_, and (*D*) *k*_cat_/*K*_m(AscA)_ of the AscA peroxidase reaction on the concentration of H_2_O_2_. The reactions were made in sodium acetate (50 mM, pH 5.0) at 25 °C. The data are presented as the average values (n = 3, independent experiments), and the error bars show the SD. The *solid lines* in panels *B* and *C* show nonlinear regression of the data to the hyperbolic function. The estimates of the values of true *k*_cat_ and *K*_m(AscA)_ are given in the plots. Because of a high error (0.06 ± 0.15 μM^−1^ s^−1^), the data point with 10 μM H_2_O_2_ is not shown in panel *D* and was also excluded from the calculation of the average value of true *k*_cat_/*K*_m(AscA)_. AscA, ascorbic acid/ascorbate.

The initial rates of AscA oxidation were consistent with the Michaelis–Menten equation (Figs. 3*E* and S10*A*). The apparent *k*_cat_ and $K_{\text{m}\left({\text{H}}_{2}{\text{O}}_{2}\right)}$ values increased with increasing [AscA] (Fig. S10, *B* and *C*), with $k_{\text{cat}}/K_{\text{m}\left({\text{H}}_{2}{\text{O}}_{2}\right)}$ being independent of [AscA] (Fig. 3*F*). The same kinetic signatures were observed also in the experiments, where the reduction of ABTS cation radical was used to measure the concentration of AscA (Fig. S7*B*) (22) instead of measuring the absorbance at 265 nm (Fig. S10). Combining the results of these two different experiment setups resulted in an average $k_{\text{cat}}/K_{\text{m}\left({\text{H}}_{2}{\text{O}}_{2}\right)}$ value of 26,900 ± 3000 M^−1^ s^−1^ for the ascorbate peroxidase reaction of *Tr*AA9A (Table 1). The dependency of initial rates of AscA oxidation on [AscA] is shown in Figure 4*A*. The apparent *k*_cat_ (Fig. 4*B*) and *K*_m(AscA)_ (Fig. 4*C*) values increased with increasing [H_2_O_2_], but their ratio, *k*_cat_/*K*_m(AscA)_ (17,100 ± 1800 M^−1^ s^−1^), was independent on [H_2_O_2_] (Fig. 4*D*). Analysis of the dependency of apparent *k*_cat_ and *K*_m(AscA)_ on [H_2_O_2_] (Fig. 4, *B* and *C*) suggested the true values of *k*_cat_ and *K*_m(AscA)_ of 2.1 s^−1^, and 140 μM, respectively. The best estimate for the true $K_{\text{m}\left({\text{H}}_{2}{\text{O}}_{2}\right)}$ was calculated as a ratio of *k*_cat_ = 2.1 ± 0.2 s^−1^ (Fig. 4*B*) and $k_{\text{cat}}/K_{\text{m}\left({\text{H}}_{2}{\text{O}}_{2}\right)}$ = 0.027 ± 0.003 μM^−1^ s^−1^ (Fig. 3*F*). This resulted in a $K_{\text{m}\left({\text{H}}_{2}{\text{O}}_{2}\right)}$ value of 78 ± 8.6 μM. However, these figures must be treated with caution because the highest [AscA] applicable in the experiments (100 μM) was not sufficiently saturating (Fig. 4*A*).

### Theoretical analysis of the H_2_O_2_-driven catalysis

**Figure 5:**
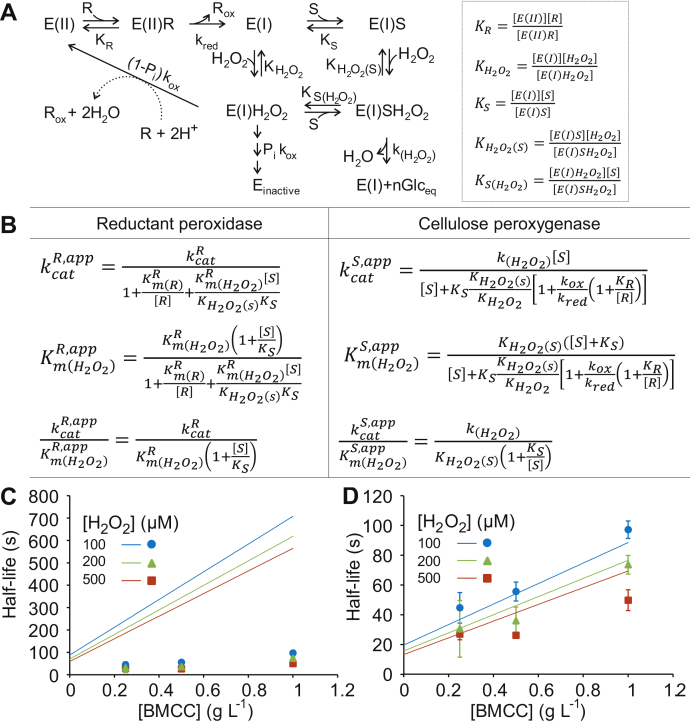
**Theoretical analysis of the H**_**2**_**O**_**2**_-**driven catalysis.***A*, kinetic mechanism of the combined cellulose peroxygenase and reductant peroxidase reactions of LPMO. E(II), E(I), and E_inactive_ stand for the LPMO-Cu^II^, LPMO-Cu^I^ redox states, and inactive enzyme, respectively. R is the reductant, and S is the cellulose substrate. The oxidative cleavage of cellulose results in the formation of *n* soluble glucose equivalents. One electron oxidation of the reductant (leading to R_ox_) takes place in the reactions of the reductant with E(II) and E(I)H_2_O_2_. Because it is not known whether the oxidation of R by hydroxyl radicals formed upon homolytic cleavage of H_2_O_2_ in the E(I)H_2_O_2_ complex (route shown with *dashed arrow*) is enzyme dependent, the rate of this step was taken independent of the [R] in deriving rate equations. In the case of AscA that was used in this study, R is ascorbate (Asc^−^) and R_ox_ is ascorbyl radical (Asc•). Two Asc• combine to give AscA and dehydroascorbate (not shown). Because the exact chemistry of enzyme inactivation is not known, this route is shown with consecutive *arrows*. The equilibrium dissociation constants used for deriving the rate equations are defined in the *right side* of the panel. *B*, equations for apparent kinetic parameters of the reductant peroxidase and cellulose peroxygenase reactions. For the equations of true parameters of the reductant peroxidase reaction in terms of the constants defined in panel *A*, refer Equations S7–S9. *C* and *D*, measured and predicted half-lives (*t*_(1/2)_) of *Tr*AA9A. The scatter points (the same data are shown in both panels) show the experimentally measured values of *t*_(1/2)_ (calculated from the *k*_obs_ values in Fig. 2*C* according to *t*_(1/2)_ = ln2/*k*_obs_). The concentrations of H_2_O_2_ are shown on the plot. The data are presented as the average values (n = 3, independent experiments), and the error bars show the SD. The *solid lines* show predicted *t*_(1/2)_ values that were calculated using Equation 3. The values of the parameters used in calculations were the following: (*C*) $k_{\text{cat}}^{\text{R}}=2.1\;{\text{s}}^{-1}$, *P*_i_ = 0.0072, $K_{\text{m}\left({\text{H}}_{2}{\text{O}}_{2}\right)}^{\text{R}}$ = 78 μM, $K_{{\text{H}}_{2}{\text{O}}_{2}\left(\text{S}\right)}$ = 30 μM ($K_{\text{m}\left({\text{H}}_{2}{\text{O}}_{2}\right)}$ for the cellulose peroxygenase reaction in Table 1), $K_{\text{m}\left(\text{R}\right)}^{\text{R}}$ = 140 μM (from Fig. 4*C*), and *K*_s_ = 0.25 g l^−1^. Because the results of the binding experiments in the presence of AscA came with high uncertainty (Fig. S5), the estimate of *K*_s_ was taken as the half-saturating concentration of BMCC for the apparent *k*_cat_ of the cellulose peroxygenase reaction in Fig. S4*B*. The concentration of the reductant ([R]) was set to 1000 μM in calculations. *D*, the same as in panel *C*, but the calculations were made using 4.5-fold higher *P*_i_ (*P*_i_ = 0.0324) and 2-fold higher *K*_s_ (*K*_s_ = 0.5 g l^−1^). AscA, ascorbic acid/ascorbate; BMCC, bacterial microcrystalline cellulose; LPMO, lytic polysaccharide monooxygenase.

Figure 5*A* shows a possible kinetic mechanism of the H_2_O_2_-driven catalysis (cellulose peroxygenase and reductant peroxidase reactions) by LPMO. Random order ternary complex and ping-pong mechanisms were used for modeling the cellulose peroxygenase and the reductant peroxidase reaction, respectively. For simplicity, the weak binding of Cu(II) enzyme forms to cellulose is omitted. Because the reactivity with O_2_ (cellulose oxygenase and reductant oxidase reaction) was negligible (Fig. S1), we have also omitted possible complexes with O_2_. The reductant peroxidase reaction was assumed to lead to the irreversible inactivation of LPMO (with a probability of *P*_i_). Chemical reactions were considered to be irreversible, and the mechanism in Figure 5*A* was solved using an equilibrium assumption for all complexes. For derivation of the rate equations, see Supporting information (Supplementary results along with Supplementary equations Equations S1–S24).

The initial rates of both, the reductant peroxidase (Equation S3) and the cellulose peroxygenase (Equation S13), reactions were consistent with the Michaelis–Menten equation with apparent parameters for H_2_O_2_ shown in Figure 5*B*. In the absence of the cellulose substrate (S), the apparent parameters of the reductant peroxidase reaction reduce to those expected for the ping-pong mechanism (Equations S12–S14), with *k*_cat_/*K*_m_ being independent of the concentration of the reagents as also observed in the experiments (Figs. 3*F* and 4*D*). Cellulose acts as a mixed-type inhibitor for the reductant peroxidase reaction, with apparent $k_{\text{cat}}/K_{\text{m}\left({\text{H}}_{2}{\text{O}}_{2}\right)}$ reduced by the competitive (*K*_s_) component of inhibition (Fig. 5*B*).

The apparent parameters of the cellulose peroxygenase reaction depend on the concentration of cellulose (Fig. 5*B*) as expected for the ternary complex mechanism. The apparent *k*_cat_ and $K_{\text{m}\left({\text{H}}_{2}{\text{O}}_{2}\right)}$ of the cellulose peroxygenase reaction increase with the increasing concentration of the reductant and the rate constant of the active site copper reduction (*k*_red_) but decrease with the increasing rate constant of the active site copper reoxidation (*k*_ox_). However, the apparent $k_{\text{cat}}/K_{\text{m}\left({\text{H}}_{2}{\text{O}}_{2}\right)}$ of the cellulolytic peroxygenase reaction is independent on the kinetic parameters of the reduction and reoxidation of the active site copper as well as on the concentration of the reductant (Fig. 5*B*).

A drawback in the application of LPMOs is the irreversible inactivation of the enzyme in the reductant peroxidase reaction. The rate of enzyme inactivation is a product (Equation S20) of the rate of the reductant peroxidase reaction and the probability of enzyme inactivation in the reductant peroxidase reaction (*P*_i_). Therefore, the apparent kinetic parameters for inactivation are the same as for the reductant peroxidase reaction, but the *k*_cat_ of the reductant peroxidase reaction $\left(k_{\text{cat}}^{\text{R}}\right)$ must be replaced with $k_{\text{cat}}^{\text{R}}P_{\text{i}}$ (in Fig. 5*B* and in Equations S4, S6, S7, S9, S12, and S14). In the presence of the reductant (R), H_2_O_2_, and cellulose (S), the half-life of LPMO (*t*_(0.5)_) is given by the following equation:

(3) $$t_{\left(0.5\right)}=\frac{\text{ln}2}{P_{\text{i}}k_{\text{cat}}^{\text{R}}}\left[\left[\text{S}\right]\left(\frac{K_{\text{m}\left({\text{H}}_{2}{\text{O}}_{2}\right)}^{\text{R}}\left(K_{{\text{H}}_{2}{\text{O}}_{2}\left(\text{S}\right)}+\left[{\text{H}}_{2}{\text{O}}_{2}\right]\right)}{K_{{\text{H}}_{2}{\text{O}}_{2}\left(\text{S}\right)}K_{\text{S}}\left[{\text{H}}_{2}{\text{O}}_{2}\right]}\right)+1+\frac{K_{\text{m}\left(\text{R}\right)}^{\text{R}}}{\left[\text{R}\right]}+\frac{K_{\text{m}\left({\text{H}}_{2}{\text{O}}_{2}\right)}^{\text{R}}}{\left[{\text{H}}_{2}{\text{O}}_{2}\right]}\right]$$

For the definition of constants, see Figure 5. The half-life of LPMO is the lowest in the absence of cellulose, but, starting from this minimum, the half-life is expected to increase linearly with an increasing cellulose concentration (Fig. 5, *C* and *D*).

## Discussion

Despite a wealth of structural and biochemical data, the quantitative kinetic studies of LPMOs are scarce. Here, we used a ^14^C-labeled cellulose substrate for the kinetic characterization of the LPMO from the cellulose-degrading model fungus *T. reesei*. *Tr*AA9A revealed high catalytic efficiency of the cellulolytic peroxygenase reaction. The $k_{\text{cat}}/K_{\text{m}\left({\text{H}}_{2}{\text{O}}_{2}\right)}$ value of 0.29 μM^−1^ s^−1^ measured on BMCC (Table 1) is well in line with the $k_{\text{cat}}/K_{\text{m}\left({\text{H}}_{2}{\text{O}}_{2}\right)}$ value of 0.27 μM^−1^ s^−1^ measured for the same enzyme on wood-derived cellulose (Avicel) using competition experiments with other H_2_O_2_-consuming enzymes (22). Unlike the competition experiments, the analyses made here also provide the values of individual *k*_cat_ and $K_{\text{m}\left({\text{H}}_{2}{\text{O}}_{2}\right)}$ (Table 1). To date, the corresponding values are available only for the chitinolytic peroxygenase reaction of *Sm*AA10A (20). *Tr*AA9A and *Sm*AA10A have similar *k*_cat_ values for the polysaccharide peroxygenase reaction, 8.5 s^−1^ and 6.7 s^−1^, respectively. However, *Sm*AA10A had about an order of magnitude lower $K_{\text{m}\left({\text{H}}_{2}{\text{O}}_{2}\right)}$ (2.8 μM and 30 μM for *Sm*AA10A and *Tr*AA9A, respectively) and, hence, higher $k_{\text{cat}}/K_{\text{m}\left({\text{H}}_{2}{\text{O}}_{2}\right)}$. Another major difference is in the apparent half-saturating concentration of AscA, which is much lower for *Sm*AA10A (around 2 μM (11) and 0.1 mM for *Sm*AA10A and *Tr*AA9A, respectively). The values of second-order rate constants of 4 × 10^4^ (34) and 6.9 × 10^3^ M^−1^ s^−1^ (33) have been reported for the reoxidation of the active site copper by H_2_O_2_ under single-turnover conditions for *Tr*AA9A and *Sm*AA10A, respectively. Thus, the requirement for higher AscA concentration in the polysaccharide peroxygenase reaction may arise from the high reductant peroxidase activity of *Tr*AA9A. Recent kinetic study of the peroxygenase activity of the *Af*AA11B with the chitotetraose substrate (*k*_cat_ = 4.0 s^−1^ and $K_{\text{m}\left({\text{H}}_{2}{\text{O}}_{2}\right)}$ = 8.9 μM) also revealed a high half-saturating concentration of the AscA (around 0.5 mM) (21). Because *Af*AA11B has unusually high AscA oxidase activity, these results also indicate to a link between the requirement for the higher AscA concentration in the peroxygenase reaction and the rate of the reoxidation of the active site copper (21).

The rate constant of 4 × 10^4^ M^−1^ s^−1^ (pH 6, 4 °C) measured for the reoxidation of *Tr*AA9A-Cu(I) by H_2_O_2_ under single-turnover conditions (34) is somewhat higher (considering the temperature difference) than the $k_{\text{cat}}/K_{\text{m}\left({\text{H}}_{2}{\text{O}}_{2}\right)}$ of 2.7 × 10^4^ M^−1^ s^−1^ found here for the AscA peroxidase reaction (Table 1). If so, the rate-limiting step of reoxidation is after the electron transfer resulting in the formation of *Tr*AA9A-Cu(II) (Jones *et al.* (34), measured the rate by following the growth of the Cu(II) signal). Because the reductant peroxidase reaction can lead to the irreversible inactivation of LPMO, an in-depth understanding of the kinetics and mechanism of this reaction is of utmost importance regarding the application of LPMOs. An experiment setup presented here (Fig. 3, *C* and *D*) allows the determination of the probability of inactivation of LPMO in the AscA peroxidase reaction (*P*_i_), which was 0.0072 (Table 1). Using the estimate of *k*_cat_ for the AscA peroxidase reaction of 2.1 s^−1^ (Table 1) and *P*_i_, one can estimate the *k*_cat_ for inactivation of *Tr*AA9A of 0.015 s^−1^. In the conditions of low cellulose and high H_2_O_2_ concentrations, the decay of the rate of the cellulose peroxygenase reaction (*k*_obs_ in Equation 1) is determined by the inactivation of LPMO (20). In this regard, we note that the *k*_obs_ values measured in the cellulose peroxygenase reaction (Fig. 2*C*) are somewhat higher than expected from the values of *P*_i_ and *k*_cat_ of the AscA peroxidase reaction (see above). The inactivation of LPMO is caused by hydroxyl radicals that form on the homolytic cleavage of O-O bond in H_2_O_2_ (16, 43). In the presence of the substrate, the hydroxyl radical is “caged” and hydrogen atom abstraction is directed toward the formation of Cu(II)-oxyl intermediate, which eventually leads to the hydroxylation of the substrate (44) and breakage of the glycosidic bond. Thus, inactivation of the enzyme in the cellulose peroxygenase reaction is unlikely. In the absence of the substrate, there is more freedom for the reactivity of hydroxyl radical and hydrogen atoms can be abstracted not only from the reductant (45) (as in Fig. 5*A*) but also from the enzyme. The latter can lead to the inactivation of enzyme. The primary targets for the oxidative damage are the copper-coordinating histidine residues, but modifications of other amino acids have also been observed (16, 43, 46). The AscA peroxidase reaction apparently assumes less contacts between the enzyme and substrate (36) than the polysaccharide peroxygenase reaction, which requires multipoint precision binding to the polysaccharide substrate (47, 48, 49, 50, 51). Therefore, it is possible that some oxidative damages that are deleterious for the polysaccharide peroxygenase reaction can be tolerated by the AscA peroxidase reaction. If so, the inactivation measured using the AscA peroxidase reaction may overestimate the performance of LPMO in the cellulose peroxygenase reaction.

The measured half-life of *Tr*AA9A increased with an increasing cellulose concentration, but the “protective effect” of cellulose was less prominent than that predicted using Equation 3 and the best estimates of the kinetic parameters (Fig. 5, *C* and *D*). Besides the *P*_i_ and *k*_cat_ of the AscA peroxidase reaction, the protective effect of cellulose is determined by the affinity of LPMO to cellulose (*K*_s_ in Equation 3), which was high for the *Tr*AA9A/BMCC system (Fig. S5). *Tr*AA9A consists of a catalytic domain and a carbohydrate-binding module, which has been shown to increase the affinity to cellulose (40). The existence of nonproductive complexes, where an enzyme is bound to cellulose only by the carbohydrate-binding module, is well-known for glycoside hydrolases (52) and has been proposed also for LPMOs (53). Because the copper active site of LPMOs in such nonproductive complexes is susceptible for inactivation, their existence reduces the protective effect of the polysaccharide. Further studies will reveal the relationships between the stability of LPMOs and possible different binding modes to the polysaccharide substrate.

For economic reasons, the enzymatic degradation of lignocellulose is performed under high dry-matter consistency (54). To maximize the stability of LPMOs, the concentration of H_2_O_2_ must be kept low (far below the $K_{\text{m}\left({\text{H}}_{2}{\text{O}}_{2}\right)}$). Under these conditions, the rate of the LPMO reaction is governed by the $k_{\text{cat}}/K_{\text{m}\left({\text{H}}_{2}{\text{O}}_{2}\right)}$ values. For *Tr*AA9A, the $k_{\text{cat}}/K_{\text{m}\left({\text{H}}_{2}{\text{O}}_{2}\right)}$ of the peroxygenase reaction is an order of magnitude higher than that for the AscA peroxidase reaction, which, in turn, was about two orders of magnitude higher than the corresponding figure for inactivation (Table 1). Furthermore, the apparent $k_{\text{cat}}/K_{\text{m}\left({\text{H}}_{2}{\text{O}}_{2}\right)}$ of the AscA peroxidase reaction and inactivation decrease with the increasing cellulose concentration (Fig. 5*B*). Thus, the values of the kinetic parameters and their dependency on cellulose concentration suggest that the flow of H_2_O_2_ through the cellulolytic peroxygenase reaction is strongly favored over side reactions. However, because the contact times required to achieve target conversion are usually in the range of 72 to 96 h (55), the stability of LPMOs is a major issue in the application of these enzymes in lignocellulose conversion (24, 56). Our results suggest that LPMOs and their variants with low efficiency of the reductant peroxidase reaction and low probability of enzyme inactivation should be more stable. Further studies are needed to reveal structural determinants of the probability of inactivation and possible trade-offs between the efficiency of the cellulose peroxygenase and the reductant peroxidase reaction.

## Experimental procedures

### Materials

2,2′-Azino-bis (3-ethylbenzothiazoline-6-sulfonic acid) diammonium salt (ABTS, lot # SLBT0759), tris-(hydroxymethyl)-aminomethane (Tris), l-ascorbic acid (AscA, lot # SLBM0850V), d-glucose, EDTA, and potassium persulfate (lot # MKCC7933) were from Sigma-Aldrich. Chelex 100 resin (50–100 mesh, sodium form) was from Bio-Rad. The H_2_O_2_ stock solution (lot # SZBG2070) was from Honeywell. D-U-^14^C-labeled glucose (300 mCi mmol^−1^, lot # 190117) was from HARTMANN ANALYTIC GmbH. Stock solutions of the sodium acetate buffer and glucose were kept over beads of Chelex 100 resin. Dilutions of the commercial H_2_O_2_ stock solution (30 wt %, 9.8 M) were prepared in water, directly before use. AscA (50 mM in water) was kept as frozen aliquots at −18 °C, and aliquots were melted directly before use. GO from *Aspergillus niger* (Sigma G6125) and horseradish peroxidase (HRP, Sigma P8375) were used as purchased. The concentration of HRP was determined by measuring the absorbance at 403 nm using a molar extinction coefficient of 102,000 M^−1^ cm^−1^ (57). The GO was dosed on weight basis. The stock solutions of HRP and GO were kept in sodium acetate (50 mM, pH 5.0) at 4 °C.

*Tr*AA9A was produced and purified as described in Kont *et al.*, 2019 (26). The purified *Tr*AA9A was saturated with copper by overnight incubation with excess CuSO_4_. The unbound copper was removed using a Toyopearl HW-40 desalting column. The concentration of *Tr*AA9A was determined by the absorbance at 280 nm using a theoretical extinction coefficient of 54,360 M^−1^ cm^−1^. ^14^C-Labeled bacterial cellulose (2.0 × 10^6^ dpm mg^−1^) was prepared by the laboratory fermentation of *Gluconobacter xylinum* (ATCC 53582) as described in (58), but the cultivation medium was supplied with uniformly ^14^C-labeled glucose (1.25 mCi g^−1^ glucose). ^14^C-Labeled BMCC was prepared using the treatment of ^14^C-labeled bacterial cellulose with HCl as described (26). The stock solutions of cellulose and *Tr*AA9A were kept in 50 mM sodium acetate (pH 5.0) at 4 °C. The water was Milli-Q ultrapure water that had been passed through a column with Chelex 100 resin. Cellulose substrates were incubated with 10 mM EDTA in 10 mM Tris, pH 8.0, overnight, followed by the removal of EDTA by thorough washing with 50 mM sodium acetate (pH 5.0) using repetitive centrifugation and resuspension steps.

### Cellulose peroxygenase reaction

*Tr*AA9A and AscA were added to ^14^C-labeled BMCC, and 30 s after the addition of AscA, the reaction was started by the addition of H_2_O_2_. At selected times, 0.18-ml aliquots were withdrawn and added to 20 μl of 1.0 M NaOH to stop the reaction. Cellulose was separated by centrifugation (3 min, 10^4^*g*), and the soluble products were quantified by measuring the radioactivity in the supernatant. The reading of the zero data point (for that, the aliquot was withdrawn before the addition of H_2_O_2_) was subtracted from the readings of the time points. The concentration of soluble products was calculated based on the radioactivity in the supernatant and the total radioactivity of cellulose in the experiment. Initial rates were calculated as the activity at 30 s. The reactions were made in 50 mM sodium acetate (pH 5.0) at 25 °C without stirring (BMCC forms a stable suspension).

### AscA peroxidase reaction

H_2_O_2_ was added to AscA, and the reaction was started by the addition of *Tr*AA9A. The oxidation of AscA was followed by the decrease in absorbance at 265 nm. The reactions were made in 50 mM sodium acetate (pH 5.0) at 25 °C without stirring in a spectrophotometer cuvette.

### Binding of *Tr*AA9A to cellulose

*Tr*AA9A (100 nM) was incubated with a nonlabeled BMCC (0–1.5 g l^−1^) for 2 min. In one series, the binding experiments were also supplied with 1 mM AscA. Cellulose was separated by centrifugation (1 min, 10^4^*g*), and the concentration of free *Tr*AA9A in the supernatant was measured by measuring its cellulose peroxygenase activity. For that, the supernatant was reacted with ^14^C-labeled BMCC (0.5 g l^−1^) and H_2_O_2_ (20 μM) for 30 s. Reactions with the supernatants from the binding experiments made without AscA were also supplied with 1 mM AscA before the measurement of cellulose peroxygenase activity. Reference reactions for 100% free *Tr*AA9A were made exactly as described above, but BMCC was omitted from the binding experiments. All reactions were made in sodium acetate (50 mM, pH 5.0) at 25 °C.

### Measurement of the stoichiometry (*n*) of cellulose peroxygenase reaction of *Tr*AA9A using *in situ* generation of H_2_O_2_ by glucose/GO reaction

For the calibration of the rate of H_2_O_2_ formation, ABTS (1 mM) was mixed with glucose (10 mM) and HRP (0.1 μM) in a spectrophotometer cuvette. The reaction was started by the addition of GO (0.05 g l^−1^), and the [H_2_O_2_] was measured by measuring the absorbance at 420 nm using the ε_420_ of 32,000 M^−1^ cm^−1^ and stoichiometry of 2ABTS^•+^/H_2_O_2_. For the cellulose peroxygenase reaction, ^14^C-labeled BMCC was mixed with glucose (10 mM), *Tr*AA9A, and AscA (1 mM). The reactions were started by the addition of GO (0.05 g l^−1^). At selected times, 0.18-ml aliquots were withdrawn and added to a 20 μl of 1.0 M NaOH to stop the reaction. Cellulose was separated by centrifugation (3 min, 10^4^*g*), and the soluble products were quantified by the radioactivity in the supernatant. The concentration of soluble products was calculated based on the radioactivity in the supernatant and the total radioactivity of cellulose in the experiment and was expressed in Glc_eq_. The reading of the zero data point (for that, the aliquot was withdrawn before the addition of GO) was subtracted from the readings of the time points. The reactions were made in 50 mM sodium acetate (pH 5.0) at 25 °C without stirring.

### Measurement of the AscA peroxidase activity of *Tr*AA9A using ABTS^•+^ for the measurement of the concentration of AscA

AscA (25, 50, or 75 μM) was mixed with *Tr*AA9A (0.125 μM), and the reaction was started by the addition of H_2_O_2_. After 120 s, an aliquot of the reaction mixture was added to the appropriately diluted mixture of ABTS/ABTS^•+^, and the concentration of AscA was determined by the decrease in absorbance at 420 nm. The volume of the aliquot of the reaction mixture was selected so that the maximum concentration of AscA in the cuvette was 10 μM. The dilution of ABTS/ABTS^•+^ was selected so that the maximum concentration of ABTS^•+^ after the addition of the reaction mixture was 25 μM. A small amount of AscA consumed in the 120-s reactions without added H_2_O_2_ was subtracted from the corresponding values measured in the presence of H_2_O_2_ before the calculation of the initial rates (always less than 20% of AscA was consumed in these experiments). The reactions were made in 50 mM sodium acetate (pH 5.0) at 25 °C. The ABTS/ABTS^•+^ stock solution was made by incubating ABTS (2.0 mM) with potassium persulfate (0.5 mM) as described (22).

## Data availability

All data are available within the article and its Supporting Information file and from the corresponding author upon reasonable request.

## Supporting information

This article contains supporting information.

## Conflict of interest

The authors declare that they have no conflicts of interest with the contents of this article.

## Supporting information

Supplementary Results, Figures S1–S10 and Equations S1–S24
