# Supplementary material for: Kinetics of H2O2-driven catalysis by a lytic polysaccharide monooxygenase from the fungus Trichoderma reesei
Source: J Biol Chem. 2021 Sep 28;297(5):101256. doi: 10.1016/j.jbc.2021.101256 (PMC8528726; doi:10.1016/j.jbc.2021.101256)
Supplement: Supplementary Results, Figures S1–S10 and Equations S1–S24 [file mmc1.pdf]

Supporting information to:

Kinetics of H_2O_2-driven catalysis by a lytic polysaccharide monooxygenase from the fungus *Trichoderma reesei*

Silja Kuusk, Priit Väljamäe*

Institute of Molecular and Cell Biology, University of Tartu, Estonia

*To whom correspondence should be addressed: Priit Väljamäe, Riia 23b – 202, 51010 Tartu, Estonia; E-mail: priit.valjamae@ut.ee

TABLE OF CONTENTS

	Page
Supplementary Figures	
Figure S1. Progress curves of the release of radioactivity from BMCC by *Tr*AA9A in the presence and absence of added H_2O_2.	2
Figure S2. Progress curves of the release of soluble products from BMCC by *Tr*AA9A at different concentrations of AscA.	3
Figure S3. Progress curves of the release of soluble products from BMCC by *Tr*AA9A at different concentrations of H_2O_2.	4
Figure S4. Dependency of initial rates and apparent parameters of cellulolytic peroxygenase reaction on [BMCC].	5
Figure S5. Binding of *Tr*AA9A to BMCC in the presence and absence of AscA.	6
Figure S6. Progress curves of the release of soluble products from BMCC by *Tr*AA9A in the experiments with H_2O_2 being supplied by glucose/glucose oxidase reaction.	7
Figure S7. Calibration curves for measurement of the concentration of AscA.	8
Figure S8. Progress curves of the oxidation of AscA by *Tr*AA9A at different concentrations of H_2O_2.	9
Figure S9. Progress curves of the oxidation of AscA by *Tr*AA9A after compensation for the AscA consumed in the experiments without the added H_2O_2.	10
Figure S10. Dependency of apparent parameters of AscA peroxidase reaction on [AscA].	12
Supplementary results	
Theoretical analysis of H_2O_2-driven catalysis	13

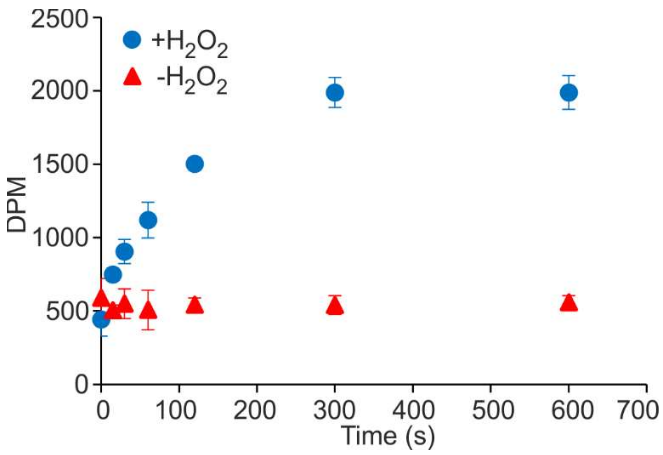

Fig. S1. Progress curves of the release of radioactivity from BMCC (1.0 g L^{-1}) by *Tr*AA9A (50 nM) in the presence (20 µM) and absence of added H_2O_2 (as indicated in the plot). Reactions were made in sodium acetate (50 mM, pH 5.0) at 25 ºC in the presence of 1 mM AscA. Data are presented as average values (*n* = 3, independent experiments) and error bars show SD.

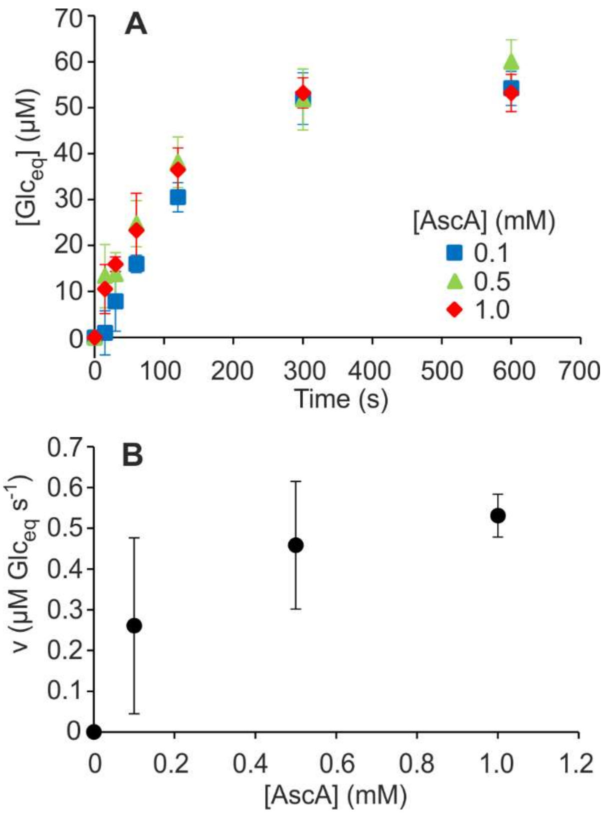

Fig. S2. (*A*) Progress curves of the release of soluble products (expressed in glucose equivalents, Glc_{eq}) from BMCC (1.0 g L^{-1}) by *Tr*AA9A (50 nM). The concentration of H_2O_2 was 20 µM and the concentration of ascorbic acid (AscA) is indicated in the plot. Reactions were made in sodium acetate (50 mM, pH 5.0) at 25 ºC. (*B*) Initial rates of the formation of soluble products (calculated based on the 30 s time points in panel A) as a function of the concentration of AscA. Data are presented as average values (n = 3, independent experiments) and error bars show SD.

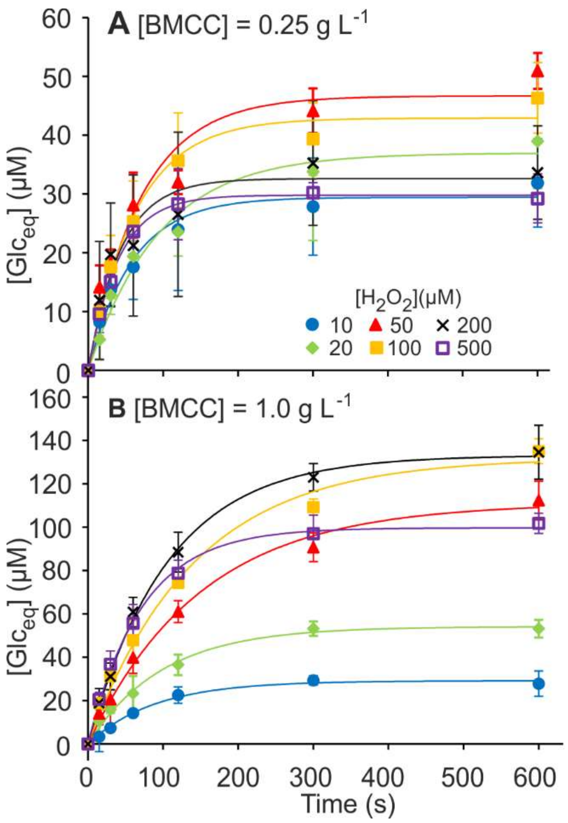

Fig. S3. Progress curves of the release of soluble products (expressed in glucose equivalents, Glc_{eq}) from BMCC by *Tr*AA9A (50 nM) at different concentrations of H_2O_2 (indicated in panel *A*). Reactions were made in sodium acetate (50 mM, pH 5.0) at 25 ºC in the presence of 1 mM AscA. Concentration of BMCC was (*A*) 0.25 g L^{-1} or (*B*) 1.0 g L^{-1} .Solid lines show non-linear regression of the data according to Eq. 1. Data are presented as average values (n = 3, independent experiments) and error bars show SD.

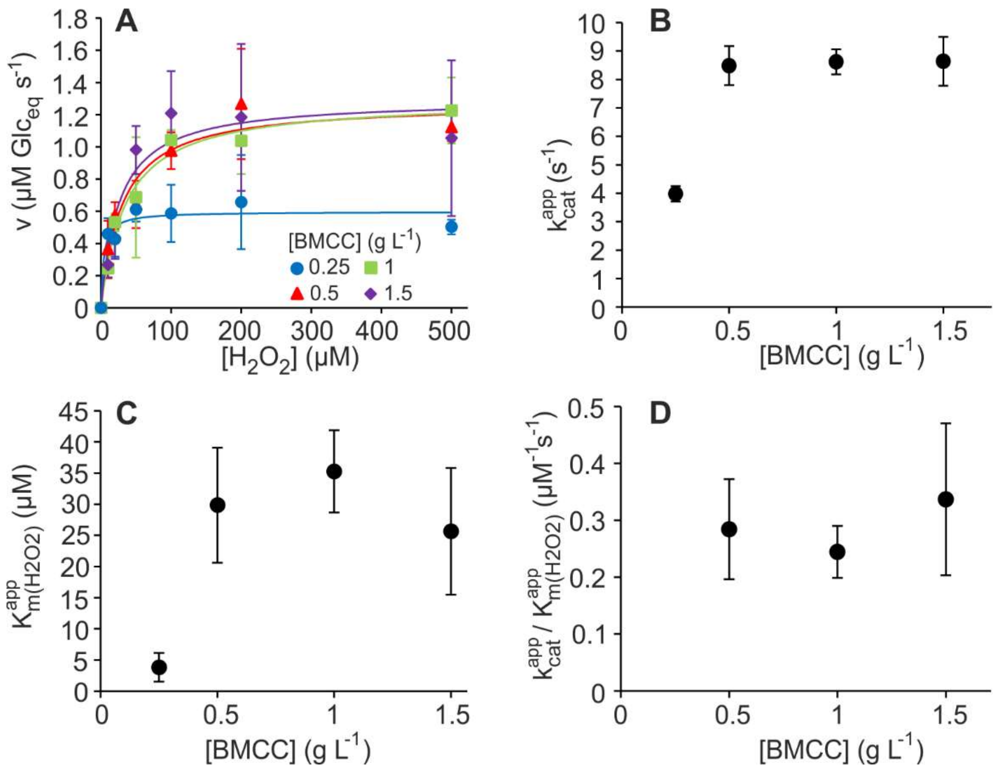

Fig. S4. (*A*) Dependency of the initial rates of the release of soluble products (calculated based on the 30 s time points) on the concentration of H_2O_2. The concentration of *Tr*AA9A was 50 nM and the concentration of BMCC is indicated in the plot. Reactions were made in sodium acetate (50 mM, pH 5.0) at 25 ºC in the presence of 1 mM AscA. Solid lines show non-linear regression of the data according to the Michaelis-Menten equation. Dependency of apparent (*B*) k_{cat}, (*C*) $K_{m(H2O2)}$, and (*D*) $k_{cat}/K_{m(H2O2)}$ of the cellulolytic peroxygenase reaction on the concentration of cellulose. Apparent $k_{cat}/K_{m(H2O2)}$ measured at 0.25 g L^{-1} BMCC is not shown because of the high uncertainty of the apparent $K_{m(H2O2)}$ measured at this low [BMCC]. Data are presented as average values (n = 3, independent experiments) and error bars show SD.

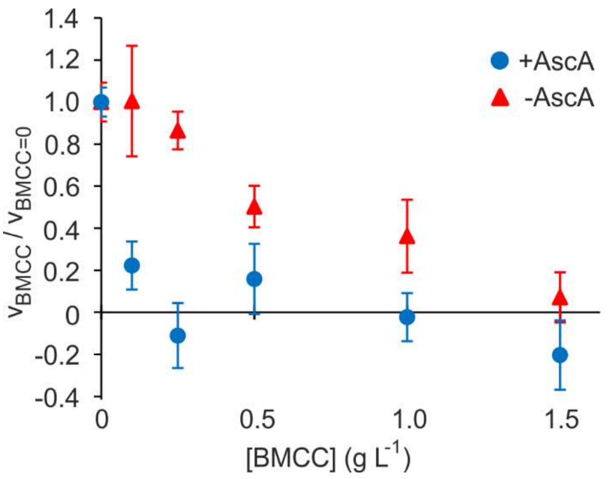

Fig. S5. Binding of *Tr*AA9A to BMCC in the presence (1 mM) and absence of AscA (as indicated in the plot). *Tr*AA9A (100 nM) was incubated with non-labeled BMCC (at concentration indicated in the x-axis) for 2 min. Cellulose was separated by centrifugation and the concentration of free *Tr*AA9A in the supernatant was measured by measuring its peroxygenase activity. v_{BMCC} is the rate of the release of soluble products from ^{14}C-labeled BMCC by the supernatants from the binding experiments with BMCC. $v_{BMCC=0}$ is the rate of the reference reaction for 100% cellulose-free *Tr*AA9A obtained from the experiments without BMCC in the binding experiment. All reactions were made in sodium acetate (50 mM, pH 5.0) at 25 ºC. Data are presented as average values (n = 3, independent experiments) and error bars show SD. Negative $v_{BMCC}/v_{BMCC=0}$ values are caused by the lower radioactivity readings in the peroxygenase assay compared to the background radioactivity readings.

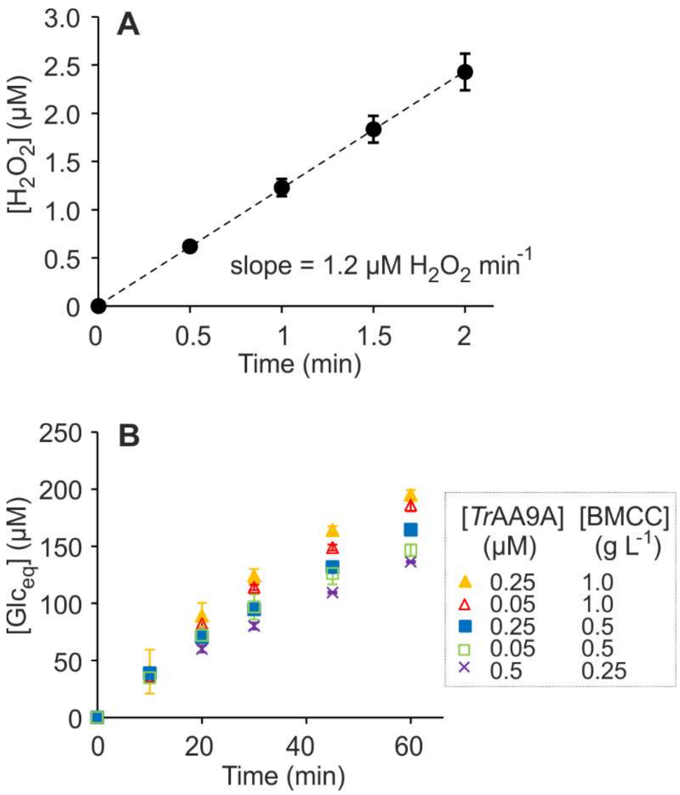

Fig. S6. (*A*) Progress curves of the formation of H_2O_2 in the glucose (10 mM)/ glucose oxidase (GO, 0.05 g L^{-1}) reaction. The reactions were made in sodium acetate (50 mM, pH 5.0) at 25 ºC. Data are presented as average values (n = 6, independent experiments) and error bars show SD. The solid line shows linear regression of the data. (*B*) Progress curves of the release of soluble products (expressed in glucose equivalents, Glc_{eq}) from BMCC by *Tr*AA9A in the experiments with H_2O_2 being supplied by glucose/glucose oxidase reaction. Reactions were made in sodium acetate (50 mM, pH 5.0) at 25 ºC and contained glucose (10 mM), GO (0.05 g L^{-1}), and AscA (1 mM). Concentrations of *Tr*AA9A and BMCC are indicated in the plot. Data are presented as average values (n = 3, independent experiments) and error bars show SD.

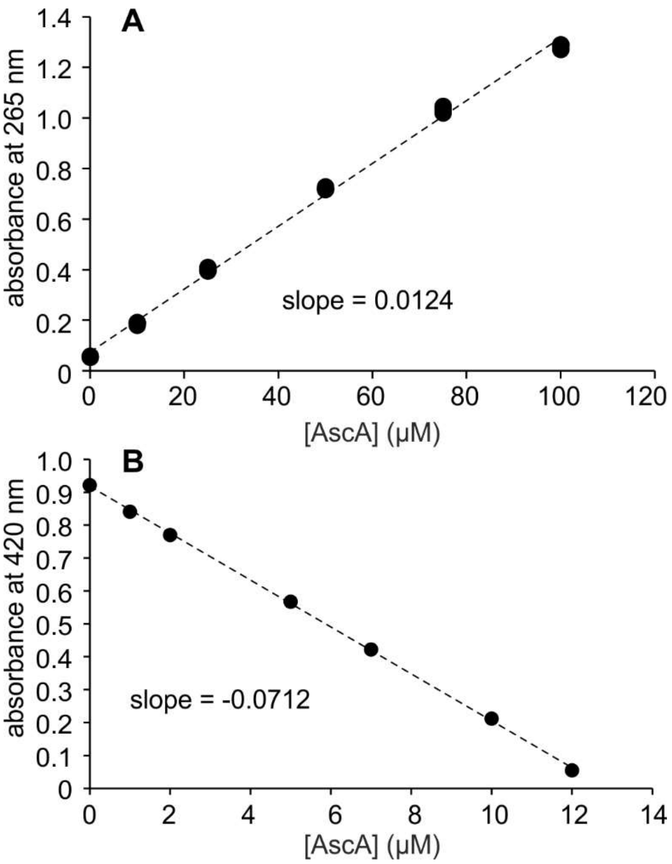

Fig. S7. Representative calibration curves for the measurement of the concentration of AscA using (*A*) absorbance at 265 nm or (*B*) reduction of the ABTS cation radical ($ABTS^{\bullet+}$). Solid lines show linear regression of the data. Note that, since radicals resulting from single electron oxidation of AscA combine to give a dehydroascorbate and ascorbic acid the loss of absorbance at 265 nm reflects the two electron oxidation of AscA.

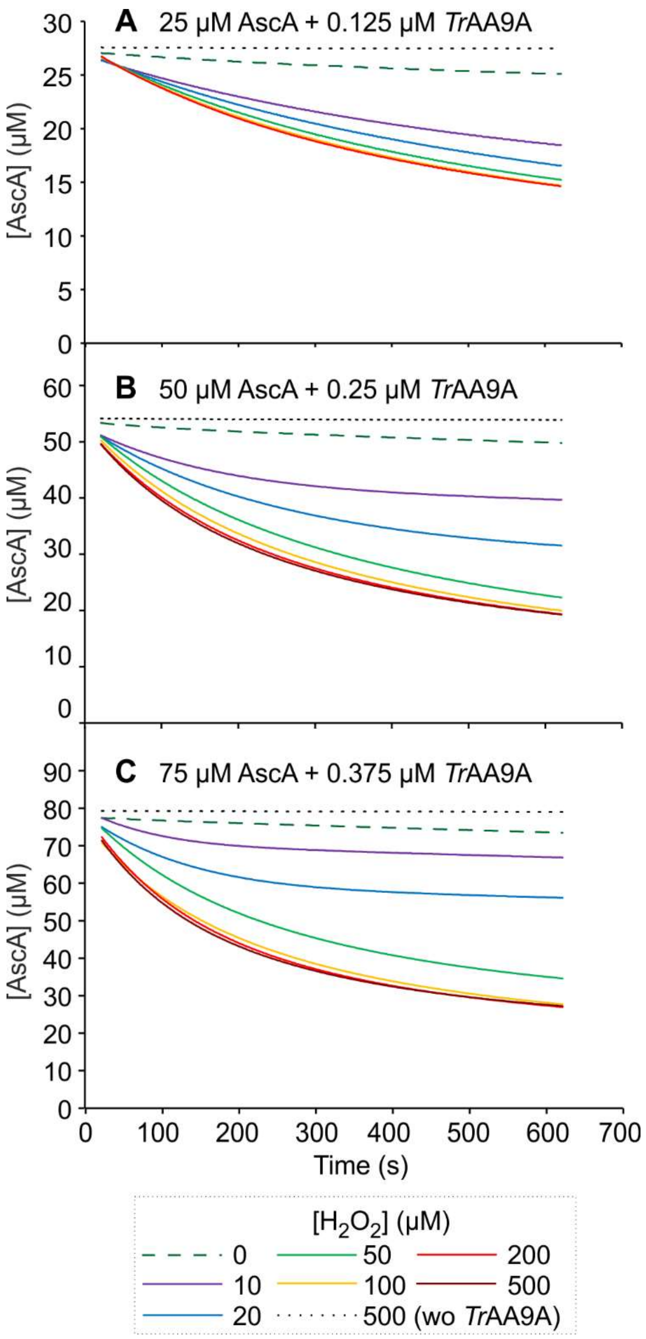

Fig. S8. Progress curves of the oxidation of AscA by *Tr*AA9A at different concentrations of H_2O_2 (indicated below the figure). The concentration of AscA and *Tr*AA9A was varied but their ratio was kept 200/1. [AscA]/[*Tr*AA9A] was (*A*) 25 µM/0.125 µM, (*B*) 50 µM/0.25 µM or (*C*) 75 µM/0.375 µM. The reactions were made in sodium acetate (50 mM, pH 5.0) at 25 ºC and the concentration of AscA was measured by measuring the absorbance at 265 nm. Traces show average values (n = 2, independent experiments). SD are not shown for clarity.

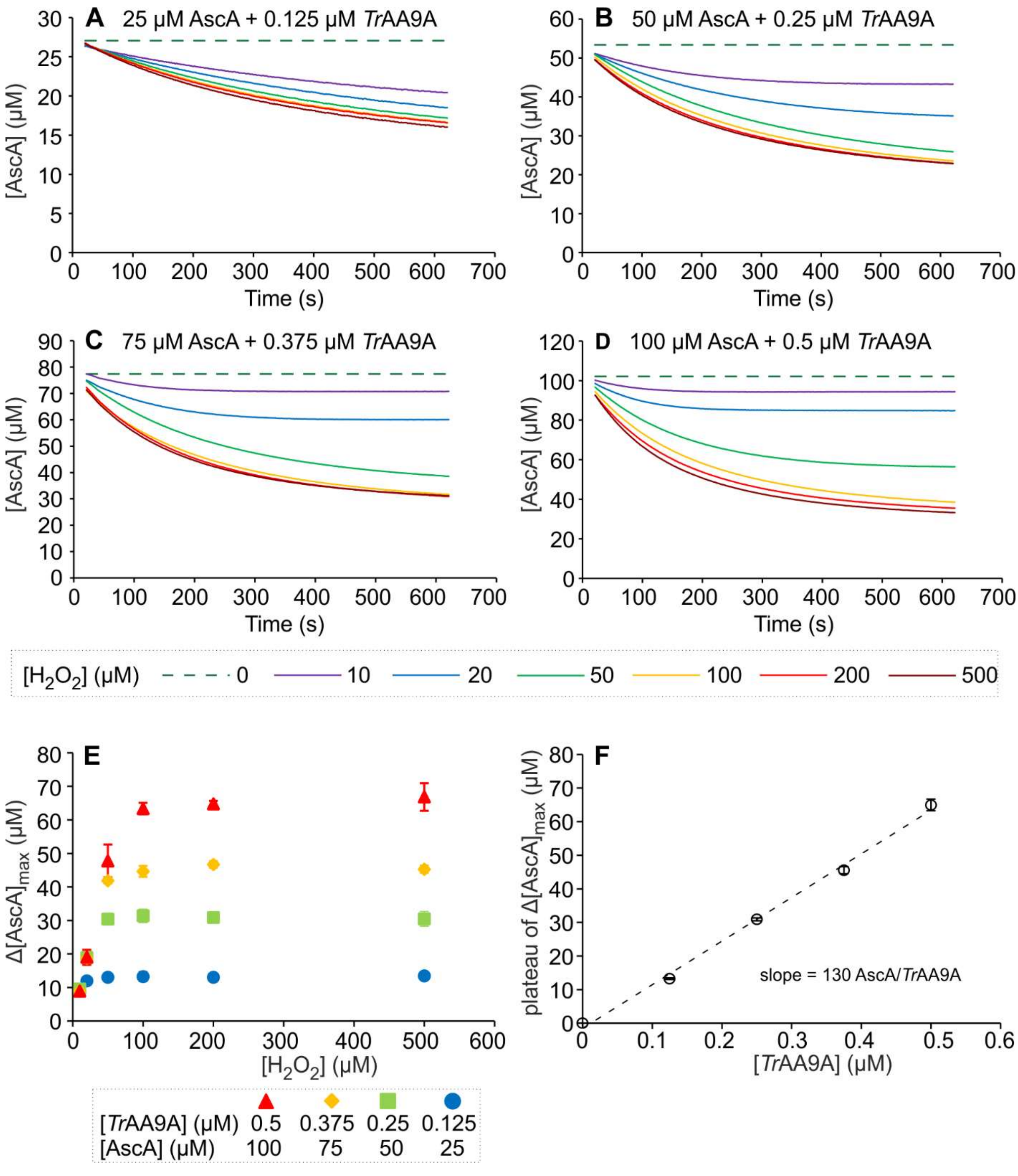

Fig. S9. (*A-D*) Progress curves of the oxidation of AscA by *Tr*AA9A at different concentrations of H_2O_2 (indicated below the panels *C-D*) after compensation for the AscA consumed in the experiments without the added H_2O_2. The concentrations of AscA and *Tr*AA9A were varied but their ratio was kept 200/1. The concentrations of AscA and *Tr*AA9A were (*A*) 25 µM and 0.125 µM, (*B*) 50 µM and 0.25 µM, (*C*) 75 µM and 0.375 µM or (*D*) 100 µM and 0.5 µM, respectively. The reactions were made in sodium acetate (50 mM, pH 5.0) at 25 ºC and the concentration of AscA was measured by measuring the absorbance at 265

nm. Traces show average values (n = 2, independent experiments). SD are not shown for clarity. For the original (non-compensated) time curves see *SI Appendix*, Fig. S8 and Fig. 2*A* of the main article. (*E*) Dependency of $\Delta[AscA]_{max}$ on the concentration of H_2O_2. The $\Delta[AscA]_{max}$ values were derived from non-linear regression of the progress curves on panels *A-D* according to Eq. 2. The concentrations of AscA and *Tr*AA9*A* are defined below the panel. (*F*) Dependency of the plateau value of $\Delta[AscA]_{max}$ (found from the data in panel *E* as an average $\Delta[AscA]_{max}$ at the concentrations of H_2O_2 100, 200, and 500 µM) on the concentration of *Tr*AA9A. Solid line shows linear regression of the data.

Note that the compensation for the AscA oxidized in the experiments without added H_2O_2 assumes that (i) the oxidation of AscA in the experiments with- and without added H_2O_2 is independent, and (ii) the rate of the oxidation of AscA in the experiments without added H_2O_2 is not dependent on the concentration of AscA. While the first assumption may be plausible, at least under certain conditions (e.g the oxidation of AscA in the experiments without added H_2O_2 is caused by the trace amounts of free copper in the reaction system), the plausibility of the second assumption is less obvious. For this reason, the compensations used in Fig. S9 are better justified in the series with low concentrations of H_2O_2, where the concentration of residual AscA is higher.

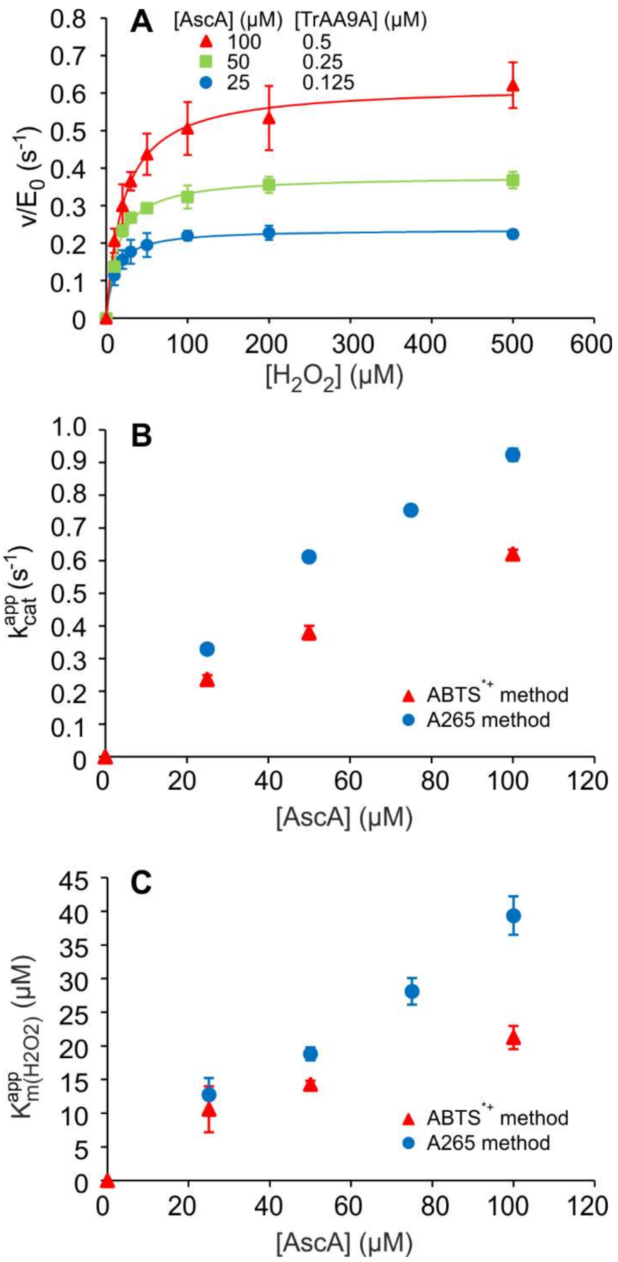

Fig. S10. (*A*) Dependency of the initial rates of the oxidation of AscA (divided to the concentration of *Tr*AA9A) on the concentration of H_2O_2. The concentrations of *Tr*AA9A and AscA are indicated in the plot. The concentration of AscA was measured using the reduction of ABTS cation radical ($ABTS^{\bullet+}$). The solid lines show non-linear regression of the data according to the Michaelis-Menten equation. Dependency of apparent (*B*) k_{cat} or (*C*) $K_{m(H2O2)}$ of the AscA peroxidase reaction on the concentration of AscA in the experiments with two different methods used for the measurement of the concentration of AscA (as indicated in the plot). The reactions were made in sodium acetate (50 mM, pH 5.0) at 25 ºC. Data are presented as average values (n = 3, independent experiments) and error bars show SD.

Theoretical analysis of H_2O_2-driven catalysis

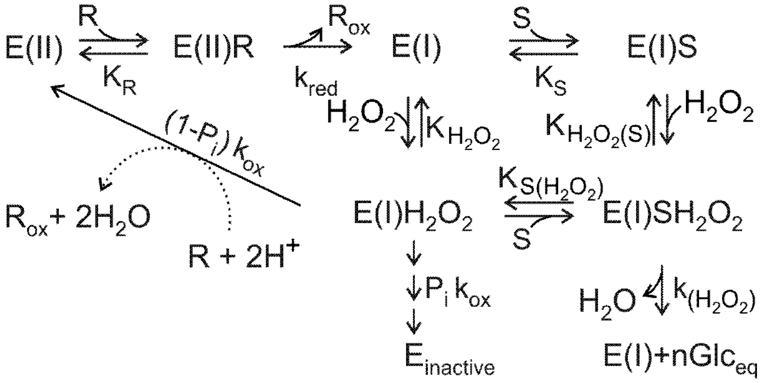

Scheme S1.

Scheme S1 (the same as in Fig. 5*A* of the main article) shows a possible kinetic mechanism of the H_2O_2-driven catalysis (cellulose peroxygenase and reductant peroxidase reactions) by LPMO. Random order ternary complex mechanism was used for modeling the cellulose peroxygenase reaction. For the reductant peroxidase reaction, we used ping-pong mechanism. For simplicity the weak binding of Cu(II) enzyme forms to cellulose is omitted. Since the reactivity with O_2 (the cellulose monooxygenase and reductant oxidase reactions) was negligible (Fig. S1) we have also omitted possible complexes with O_2. The reductant peroxidase reaction was assumed to lead to the irreversible inactivation of LPMO (with a probability of P_i). Since it is not known whether the oxidation of R by hydroxyl radicals formed upon homolytic cleavage of H_2O_2 in $E(I)H_2O_2$ complex (route shown with dashed arrow) is enzyme dependent, the rate of this step was taken independent of the [R] in deriving rate equations. Chemical reactions were considered to be irreversible and the mechanism in Scheme S1 was solved using an equilibrium assumption for all complexes. The equilibrium dissociation constants are defined as follows:

$$K_R = \frac{[E(II)][R]}{[E(II)R]} \quad K_{H_2O_2} = \frac{[E(I)][H_2O_2]}{[E(I)H_2O_2]} \quad K_S = \frac{[E(I)][S]}{[E(I)S]} \quad K_{H_2O_2(S)} = \frac{[E(I)S][H_2O_2]}{[E(I)SH_2O_2]}$$

$$K_{S(H_2O_2)} = \frac{[E(I)H_2O_2][S]}{[E(I)SH_2O_2]}$$

At steady-state the redox-state of the enzyme is constant meaning that the rates of the routes leading to the oxidation and reduction of the active site copper must be equal. So we can write $(1\text{-}P_i)k_{ox}[E(I)H_2O_2] + P_ik_{ox}[E(I)H_2O_2] = k_{red}[E(II)R]$ and:

$$[E(II)] + [E(II)R] = \frac{k_{ox}}{k_{red}} [E(I)H_2O_2] \left(1 + \frac{K_R}{[R]}\right) \qquad \text{(S1)}$$

Note that the redox-state of copper in inactive enzyme is not known but here we assume it being Cu(II). However, since $P_i << 1$, the rate of enzyme inactivation has not significant contribution to the rate of reductant peroxidase reaction.

Reductant peroxidase reaction.

The rate of the reductant peroxidase reaction (v^R_{ox}) is defined by the rates of the chemical reactions of $E(I)H_2O_2$ complex (Scheme S1):

$$v_{ox}^{R} = -\frac{d[AscA]}{dt} = (1-P_i)k_{ox}[E(I)H_2O_2] + P_i k_{ox}[E(I)H_2O_2] = k_{ox}[E(I)H_2O_2] \quad \text{(S2)}$$

Since the AscA peroxidase reaction followed 1/1 stoichiometry, the rate of AscA disappearance equals to the rate of H_2O_2 consumption in the peroxidase reaction. Solving the mechanism in Scheme S1 results in the rate equation in the form of the Micahelis-Menten equation.

$$v_{ox}^{R} = \frac{E_0 k_{cat}^{R,app}[H_2O_2]}{K_{m(H_2O_2)}^{R,app}+[H_2O_2]} \quad \text{(S3)}$$

The apparent kinetic parameters of the reductant peroxidase reaction depend on the concentrations of cellulose substrate ([S]) and reductant ([R]) as follows.

$$k_{cat}^{R,app} = \frac{k_{cat}^{R}}{1+\frac{K_{m(R)}^{R}}{[R]}+\frac{K_{m(H_2O_2)}^{R}[S]}{K_{H_2O_2(s)}K_S}} \quad \text{(S4)}$$

$$K_{m(H_2O_2)}^{R,app} = \frac{K_{m(H_2O_2)}^{R}\left(1+\frac{[S]}{K_S}\right)}{1+\frac{K_{m(R)}^{R}}{[R]}+\frac{K_{m(H_2O_2)}^{R}[S]}{K_{H_2O_2(s)}K_S}} \quad \text{(S5)}$$

$$\frac{k_{cat}^{R,app}}{K_{m(H_2O_2)}^{R,app}} = \frac{k_{cat}^{R}}{K_{m(H_2O_2)}^{R}\left(1+\frac{[S]}{K_S}\right)} \quad \text{(S6)}$$

Using the rate- and equilibrium constants defined in Scheme S1 the true parameters of the reductant peroxidase reaction are defined as follows:

$$k_{cat}^{R} = \frac{k_{ox}k_{red}}{k_{ox}+k_{red}} \quad \text{(S7)}$$

$$K_{m(H_2O_2)}^{R} = K_{H_2O_2}\frac{k_{red}}{k_{ox}+k_{red}} \quad \text{(S8)}$$

$$\frac{k_{cat}^{R}}{K_{m(H_2O_2)}^{R}} = \frac{k_{ox}}{K_{H_2O_2}} \quad \text{(S9)}$$

$$K_{m(R)}^{R} = K_R\frac{k_{ox}}{k_{ox}+k_{red}} \quad \text{(S10)}$$

$$\frac{k_{cat}^{R}}{K_{m(R)}^{R}} = \frac{k_{red}}{K_{H_2O_2}} \quad \text{(S11)}$$

In the absence of cellulose substrate (S) the apparent parameters of the reductant peroxidase reaction reduce to those expected for the ping-pong mechanism (apparent k_{cat}/K_m is independent on the concentrations of the substrates):

$$k_{cat}^{R,app} = \frac{k_{cat}^{R}[R]}{[R]+K_{m(R)}^{R}} \quad \text{(S12)}$$

$$K_{m(H_2O_2)}^{R,app} = \frac{K_{m(H_2O_2)}^{R}[R]}{[R]+K_{m(R)}^{R}} \quad \text{(S13)}$$

$$\frac{k_{cat}^{R,app}}{K_{m(H_2O_2)}^{R,app}} = \frac{k_{cat}^{R}}{K_{m(H_2O_2)}^{R}} \quad \text{(S14)}$$

Cellulolytic peroxygenase reaction.

The rate of the cellulolytic peroxygenase reaction (v^S_{ox}) is defined by the rate of chemical reaction of $E(I)SH_2O_2$ ternary complex in Scheme S1:

$$v^S_{ox} = \frac{d[Glc_{eq}]}{ndt} = k_{(H_2O_2)}[E(I)SH_2O_2] \qquad \text{(S15)}$$

In eq S15, n is the stoichiometry coefficient showing the number of soluble products (in glucose equivalents, Glc_{eq}) released per on H_2O_2 consumed in the peroxygenase reaction (for *Tr*AA9A/BMCC system n = 3.0, see main article). Since the cellulose peroxygenase reaction follows the stoichiometry of one glycosidic bond cleavage per one molecule of H_2O_2 the v^S_{ox} equals to the rate of H_2O_2 consumption in the peroxygenase reaction. Solving the mechanism in Scheme S1 results in the rate equation in the form of the Micahelis-Menten equation.

$$v^S_{ox} = \frac{E_0 k^{S,app}_{cat}[H_2O_2]}{K^{S,app}_{m(H_2O_2)} + [H_2O_2]} \qquad \text{(S16)}$$

The apparent kinetic parameters of the cellulolytic peroxygenase reaction depend on the concentrations of cellulose substrate ([S]) and reductant ([R]) as follows.

$$k^{S,app}_{cat} = \frac{k_{(H_2O_2)}[S]}{[S] + K_S \frac{K_{H_2O_2(S)}}{K_{H_2O_2}}\left[1 + \frac{k_{ox}}{k_{red}}\left(1 + \frac{K_R}{[R]}\right)\right]} \qquad \text{(S17)}$$

$$K^{S,app}_{m(H_2O_2)} = \frac{K_{H_2O_2(S)}([S] + K_S)}{[S] + K_S \frac{K_{H_2O_2(S)}}{K_{H_2O_2}}\left[1 + \frac{k_{ox}}{k_{red}}\left(1 + \frac{K_R}{[R]}\right)\right]} \qquad \text{(S18)}$$

$$\frac{k^{S,app}_{cat}}{K^{S,app}_{m(H_2O_2)}} = \frac{k_{(H_2O_2)}}{K_{H_2O_2(S)}\left(1 + \frac{K_S}{[S]}\right)} \qquad \text{(S19)}$$

Inactivation of enzyme.

The rate of enzyme inactivation in the reductant peroxidase reaction (v_i) is given by v^R_{ox} and the probability of enzyme inactivation in the reductant peroxidase reaction (P_i).

$$v_i = P_i v^R_{ox} = P_i k_{ox}[E(I)H_2O_2] \qquad \text{(S20)}$$

The apparent parameters of Michaelis-Menten equation for enzyme inactivation are exactly the same as those for the reductant peroxidase reaction, but k_{cat}^R in Eq-s S4, S6, S7, S9, S12 and S14 must be multiplied with P_i.

The half life of the enzyme in the reductant peroxidase reaction ($t_{(0.5)}$) is given by

$$t_{(0.5)} = ln2 \frac{E_0}{P_i v^R_{ox}} \qquad \text{(S21)}$$

Combining Eq. S21 with Eq-s S3 – S5 results in the equation showing the dependency of the half-life of LPMO on the concentrations of reductant (R), H_2O_2 and cellulose (S):

$$t_{(0.5)} = \frac{ln}{P_i k_{cat}^R}\left[[S]\left(\frac{K_{m(H_2O_2)}^R\left(K_{H_2O_2(S)}+[H_2O_2]\right)}{K_{H_2O_2(S)}K_S[H_2O_2]}\right)+1+\frac{K_{m(R)}^R}{[R]}+\frac{K_{m(H_2O_2)}^R}{[H_2O_2]}\right] \quad \text{(S22)}$$

It follows from Eq. 22 that the half life of LPMO is a linear function of the concentration of cellulose with the intercept:

$$Intercept = \frac{ln}{P_i k_{cat}^R}\left[1+\frac{K_{m(R)}^R}{[R]}+\frac{K_{m(H_2O_2)}^R}{[H_2O_2]}\right] \quad \text{(S23)}$$

And slope:

$$Slope = \frac{ln}{P_i k_{cat}^R}\left[\left(\frac{K_{m(H_2O_2)}^R\left(K_{H_2O_2(S)}+[H_2O_2]\right)}{K_{H_2O_2(S)}K_S[H_2O_2]}\right)\right] \quad \text{(S24)}$$
